# Decoding brain state transitions in the pedunculopontine nucleus: cooperative phasic and tonic mechanisms

**DOI:** 10.3389/fncir.2015.00068

**Published:** 2015-10-31

**Authors:** Anne Petzold, Miguel Valencia, Balázs Pál, Juan Mena-Segovia

**Affiliations:** ^1^MRC Anatomical Neuropharmacology Unit, Department of Pharmacology, University of OxfordOxford, UK; ^2^Neurosciences Area, CIMA, Universidad de NavarraPamplona, Spain; ^3^IdiSNA, Navarra Institute for Health ResearchPamplona, Spain; ^4^Department of Physiology, Faculty of Medicine University of DebrecenDebrecen, Hungary; ^5^Center for Molecular and Behavioral Neuroscience, Rutgers UniversityNewark, NJ, USA

**Keywords:** cholinergic neurons, phasic, arousal, network activity, oscillations, brainstem

## Abstract

Cholinergic neurons of the pedunculopontine nucleus (PPN) are most active during the waking state. Their activation is deemed to cause a switch in the global brain activity from sleep to wakefulness, while their sustained discharge may contribute to upholding the waking state and enhancing arousal. Similarly, non-cholinergic PPN neurons are responsive to brain state transitions and their activation may influence some of the same targets of cholinergic neurons, suggesting that they operate in coordination. Yet, it is not clear how the discharge of distinct classes of PPN neurons organize during brain states. Here, we monitored the *in vivo* network activity of PPN neurons in the anesthetized rat across two distinct levels of cortical dynamics and their transitions. We identified a highly structured configuration in PPN network activity during slow-wave activity that was replaced by decorrelated activity during the activated state (AS). During the transition, neurons were predominantly excited (phasically or tonically), but some were inhibited. Identified cholinergic neurons displayed phasic and short latency responses to sensory stimulation, whereas the majority of non-cholinergic showed tonic responses and remained at high discharge rates beyond the state transition. *In vitro* recordings demonstrate that cholinergic neurons exhibit fast adaptation that prevents them from discharging at high rates over prolonged time periods. Our data shows that PPN neurons have distinct but complementary roles during brain state transitions, where cholinergic neurons provide a fast and transient response to sensory events that drive state transitions, whereas non-cholinergic neurons maintain an elevated firing rate during global activation.

## Introduction

Brain state transitions between sleep and wakefulness produce robust changes in the spike rate and pattern of distinct classes of neurons across the brain. Neurons in the brainstem, midbrain and basal forebrain are particularly responsive to brain state changes, tuning their activity to produce a homeostatic balance in the sleep-wake cycle (Aston-Jones and Bloom, 1981; Fornal et al., 1985; Steriade et al., 1991; Sherin et al., 1996; Szymusiak et al., 1998; Steininger et al., 1999; Lee et al., 2005). This has led to the notion that some of these structures causally influence global states (for reviews, see Saper et al., 2005; Lee and Dan, 2012). Pharmacological and optogenetic experiments have shown that some neuromodulatory neuronal groups are able to *awaken* the brain over the course of several seconds (Adamantidis et al., 2007; Carter et al., 2010; Irmak and de Lecea, 2014), supporting the idea of a coordinated, albeit arguably redundant, modulation of brain states by ascending neuromodulatory neurons. While a causal relationship has been established for some of these neuronal groups, less is known about the network dynamics in which they operate. Interestingly, most sleep/wake-related neuromodulatory neurons are embedded within a network of neurochemically-distinct neurons (e.g., glutamatergic and GABAergic) whose operational features are similar to the neuromodulatory circuits that contain them. Such is the case of the pedunculopontine nucleus (PPN), a neurochemically heterogeneous brainstem structure whose cholinergic neurons have been associated with modulation of brain states.

Early theories of the role of PPN cholinergic neurons in wakefulness arose from experiments showing that the firing of neurons in a cholinergic-rich region of the brainstem (PPN) was closely related to the cortical activated states (AS; i.e., wakefulness and REM sleep; Steriade et al., 1990). In addition, electrical stimulation of the PPN region led to a fast and robust activation of the electroencephalogram and the induction of fast frequency oscillations in the gamma range (25–80 Hz; Steriade et al., 1991). Further experiments supported a role for cholinergic transmission in the modulation of fast frequency oscillations in the cortex (Mena-Segovia et al., 2008), presumably through the activation of thalamic neurons (Paré et al., 1990; Ye et al., 2009). Thus, cholinergic neurons seem to contribute to the modulation of the waking state.

The non-cholinergic neuronal population of the PPN is composed of glutamatergic and GABAergic neurons (Wang and Morales, 2009), and is far larger and more heterogeneous than the cholinergic population in terms of their neurochemical markers (Martinez-Gonzalez et al., 2012) and their firing properties (Ros et al., 2010; Boucetta et al., 2014). Notably, non-cholinergic neurons project to some of the same areas that cholinergic neurons innervate (Mena-Segovia et al., 2008; Barroso-Chinea et al., 2011; Dautan et al., 2014) and their activity is also modulated by brain states (Ros et al., 2010; Boucetta et al., 2014), suggesting that they can differentially influence the activity of their common targets as a function of the brain state. Furthermore, non-cholinergic neurons are intermingled with cholinergic neurons throughout the whole extent of the PPN (Mena-Segovia et al., 2009; Wang and Morales, 2009), and because they cannot be set apart on the basis of their electrophysiological properties (i.e., spike rate, spike pattern or action potential duration), it is likely that early reports (e.g., El Mansari et al., 1989; Steriade et al., 1990; Sakai, 2012) may have indistinctly recorded cholinergic and non-cholinergic and used the data from different phenotypes to build the prevailing model of cholinergic function during AS.

In order to investigate the contributions of different PPN neurons to brain states and their transition, we used high-density electrophysiological recordings in the urethane-anesthetized rat. We analyzed the network activity in the PPN and its correlation with global brain states. Then we used the juxtacellular labeling method to detect the neurochemical composition of the recorded neurons and to correlate this with the network properties. Finally, we recorded cholinergic and non-cholinergic neurons *in vitro* to identify their physiological properties and to complement the findings from the *in vivo* recordings. Our results illustrate different but complementary modes of operation for cholinergic and non-cholinergic neurons during brain state transitions.

## Materials and Methods

### Animals

For the *in vivo* experiments, male adult (250–350 g) Sprague-Dawley rats were used (*n* = 8; Charles River, Margate, UK). Rats were maintained on a 12 h light cycle (lights on 07:00) and had *ad libitum* access to water and food. For the *in vitro* experiments, 10–13 days old mice of both sexes expressing tdTomato fluorescent proteins associated to the GAD2 (*n* = 3) or choline acetyltransferase (ChAT; *n* = 6) promoter were used. In order to obtain mice expressing tdTomato fluorescent protein in a GAD2- or ChAT-dependent way, floxed-stop-tdTomato mice [(Madisen et al., 2010); JAX mice accession number 007905] were crossed with GAD2-cre [(Taniguchi et al., 2011); JAX number: 010802] or ChAT-cre (http://www.informatics.jax.org/reference/J:114556; JAX number: 006410) mouse lines, respectively. Mice were purchased from Jackson Laboratories (Bar Harbor, ME, USA) and bred in the animal house of the Department of Physiology (Debrecen). The animals were subjected to the lowest possible levels of pain and discomfort. All procedures were performed in accordance with the Society for Neuroscience policy on the use of animals in neuroscience and the Animals (Scientific Procedures) Act, 1986 (UK) and EU Directive 2010/63/EU, under the authority of a Project License approved by the Home Office and the local ethical committee of the University of Oxford, and the Committee of Animal Research of the University of Debrecen.

### *In vivo* High-Density and Juxtacellular Electrophysiological Recordings

Rats were initially anesthetized with 4% v/v isoflurane O_2_ and urethane (1.3 g/kg, i.p.), as described by Mena-Segovia et al. (2008). Supplementary doses of ketamine (30 mg/kg, i.p.) and xylazine (3 mg/kg, i.p.) were used as required. Body temperature was maintained at 38°C using a thermistor-controlled heating pad. After local skin anesthesia by a subcutaneous injection of bupivacaine (0.25% w/v; Astra), the animals were placed in a stereotaxic frame (Kopf). A cutaneous incision was made to expose the skull. Then, craniotomies were made for the electrocorticogram (ECoG; from bregma, AP: +3.0 mm; ML: 2.5 mm; corresponding to the somatic sensorimotor cortex) and its reference (above the right cerebellum). A small square craniotomy was made above the PPN (from Bregma in mm, AP: −4.8 to −6.2; ML: 0.7–1.5: DV: 6.8–8.2 ventral of the dura, at a 15° angle; Paxinos and Watson, 1986) and the dura mater was gently removed to allow the passage of either a silicon probe for high-density recordings or a glass pipette for single cell recordings (see below); the exposed brain surface was kept moist with sterile saline (0.9% NaCl) throughout the experiment. A supplementary ground for the single cell electrode was placed subcutaneously in the back of the neck. Electrocardiogram, ECoG and reflexes were closely monitored to control depth of anesthesia and ensure animals’ well-being. ECoG signals were recorded using steel screws of 1 mm in diameter juxtaposed to the dura mater, bandpass filtered (0.3–1500 Hz, 3 dB limits) and amplified (2000×; DPA-2FS filter/amplifier; Scientifica, Harpenden, UK).

High-density PPN electrophysiological recordings were obtained using 16- to 32-channel silicon probes (models A1x16 and A2x16, 10 mm; NeuroNexus Technologies, Ann Arbor, MI, USA). Each probe features 16 (one shank) or 32 (two shanks) vertically aligned contacts of ~400 μm^2^ spaced evenly at 100 μm with an impedance of 0.9–1.3 MΩ at 1000 Hz. Probes were advanced slowly into the brain under stereotaxic control using a zero-drift micromanipulator (1760–61; David Kopf Instruments), at an angle of 15° to the vertical to avoid damage to prominent blood vessels. Probe signals were referenced against a screw implanted in the skull above the contralateral cerebellum. Signals were amplified (1000–2000×) and low-pass filtered (0–6000 Hz) using computer-controlled differential amplifiers (Lynx-8; Neuralynx, Tucson, AZ, USA). Both the ECoG and probe signals were sampled at 17.5 kHz. All biopotentials were digitized on-line using a Power1401 analog-to-digital converter (Version 7; Cambridge Electronic Design, Cambridge, UK) and a personal computer running Spike2 acquisition and analysis software.

Single-unit activity in the PPN was recorded using 15–25 MΩ glass electrodes (tip diameter 1.5 μm), filled with saline solution (0.5 M NaCl) and neurobiotin (1.5% w/v, Vector Laboratories Ltd., Peterborough, UK). Glass electrode signals were amplified (10×) through the active bridge circuitry of an Axoprobe-1A amplifier (Molecular Devices Corp., Sunnyvale, CA, USA), AC-coupled, and further amplified (100×, NL-106 AC-DC Amp: Digitimer Ltd., Welwyn Garden City, UK), before being band-pass filtered (0.3 Hz-5 kHz; NL125: Digitimer) and digitized online at 17.5 kHz. Data were acquired and stored using an analog-to-digital converter (Power 1401) connected to a PC running Spike2.

Neural activity was recorded during cortical slow-wave activity (SWA) and during episodes of both spontaneous and sensory-evoked cortical AS. Such comprehensive recordings enabled us to observe neural activity during changes from SWA, which accompanies deep anesthesia and is similar to activity observed during natural (non-REM) sleep, to AS, which contains patterns of activity that are more analogous to those observed during the awake, behaving state (for review, see Steriade, 2000). Sensory-evoked stimulation was induced by a standardized pinch of the hind paw using pneumatically driven serrated forceps at a regular pressure of 183 g/mm^2^ for 15 s and produced global activation as indicated by an initial obliteration of cortical slow oscillations that was replaced by fast-frequency, low-amplitude activity in the ECoG. The nature and duration of cortical changes following the pinch varied within individual recordings and between different animals, however, eventually relaxing back to slow oscillations. No overt behavioral reaction by the animals was observed in response to the pinch.

### Juxtacellular Labeling of PPN Neurons

Following electrophysiological recordings, neurons were labeled with neurobiotin in order to examine their exact location and to identify their neurochemical properties (Pinault, 1996). Once the spontaneous firing of a neuron was recorded, a microiontophoretic current was applied (1–10 nA positive current, 200 ms duration, 50% duty cycle), while the electrode was slowly advanced towards the neuron. The optimal position of the electrode was reached once the firing pattern was robustly modulated by the current injection. This modulation of the firing was maintained for at least 2 min in order to obtain reliable labeling. The neurobiotin was then allowed to distribute throughout along neuronal processes for 5–12 h, when animals were given a lethal dose of ketamine (150 mg/kg) and upon cessation of all reflexes, were intracardially perfused with 0.05 M phosphate buffered saline (PBS), pH 7.4, followed by 300 ml of 4% w/v paraformaldehyde and 0.1% w/v glutaraldehyde in phosphate buffer (0.1 M pH 7.4). Brains were stored in PBS at 4°C until sectioning.

Individually recorded and identified neurons reported in this study were selected from previous datasets (Mena-Segovia et al., 2008; Ros et al., 2010) based on the presence of a significant response to the pinch (see below).

### *In vitro* Recordings

Experiments were performed in an artificial cerebrospinal fluid (aCSF) of the following composition (in mM): NaCl, 125; KCl, 2.5; NaHCO_3_, 26; glucose, 10; NaH_2_PO_4_, 1.25; CaCl_2_, 2; MgCl_2_, 1; myo-inositol, 3; ascorbic acid, 0.5; and sodium-pyruvate, 2, and kept at room temperature (cca. 25°C). For the slice preparation, 100 mM NaCl was replaced by sucrose (130 mM) and glycerol (60 mM; low Na aCSF). All chemicals were purchased from Sigma (St. Louis, MO, USA), unless stated otherwise. After decapitation of the mice and removal of the brain, 200 μm-thick coronal midbrain slices were prepared in ice-cold low Na aCSF using a Microm HM 650 V vibratome (Microm International GmbH, Walldorf, Germany). Brain slices were visualized with a Zeiss Axioskop microscope (Carl Zeiss AG, Oberkochen, Germany). Patch pipettes with 5 MΩ pipette resistance were fabricated, and filled with a solution containing (in mM): K-gluconate, 120; NaCl, 5; 4-(2-hydroxyethyl)-1- piperazineethanesulfonic acid (HEPES), 10; EGTA, 2; CaCl_2_, 0.1; Mg-ATP, 5; Na_3_-GTP, 0.3; Na_2_- phosphocreatinine, 10; biocytin, 8. Whole-cell patch-clamp recordings were performed using an Axopatch 200A amplifier (Molecular Devices, Union City, CA, USA). Data acquisition was achieved using the Clampex 10.0 software (Molecular Devices, Union City, CA, USA), while data analysis was performed using the Clampfit 10.0 (Molecular Devices) program. For calculating input resistance, a 1-s-long hyperpolarizing current injection with 30 pA amplitude was applied in current clamp mode. In order to observe long-term frequency adaptation of the different cell types of the PPN, 10-s-long depolarizing current injections were applied with 5 pA increments. To exclude differences caused by activation of A-current (and other conductances depending on holding current), the holding potential was kept at −60 mV. For the traces used for statistics analysis of firing frequency, the membrane potential was depolarized to −40 mV. Due to the difference in input resistance of individual neurons, current injections between 20–75 pA were applied.

### Histological Processing and Immunohistochemistry

Rat brains were sectioned at 50 μm in the parasagittal plane on a vibratome (Leica). Silicon probe recording locations were histologically verified in all animals. Previous to the recordings, the silicon probes were coated with the red fluorescent dye 1,1^′^-dioctadecyl-3,3,3^′^,3^′^-tetramethylindocarbocyanine perchlorate (DiI; Invitrogen, Carlsbad, CA, USA), by immersion of the probe in a 80 mg/ml solution (in 50/50% acetone/methanol) under microscopic control, as described previously (Magill et al., 2006). Using light microscopy, DiI labeling was confirmed to be present in the PPN region, and only recordings where the majority of the probe contacts were within the PPN borders were considered.

In order to identify the neurochemical profile and location of juxtacellularly-labeled neurons, neurobiotin was revealed by incubation with CY3-conjugated streptavidin (1:1000; Jackson ImmunoResearch Laboratories, Inc., USA) in PBS containing 0.3% v/v Triton X-100. The presence of ChAT, the synthetic enzyme for acetylcholine, was revealed by incubation in goat anti-ChAT antibodies (1:500, Chemicon, USA), followed by Alexa 488-conjugated donkey anti-goat antibodies (1:1000, Jackson ImmunoResearch Laboratories, Inc.). Sections were mounted on slides for imaging with a conventional epifluorescence microscope (DMRB: Leica Microsystems GmbH, Wetzlar, Germany) or a laser-scanning confocal fluorescence microscope (LSM510: Karl Zeiss AG, Oberkochen, Germany). ChAT labeling was evaluated for presence of immunoreactivity in the cytoplasm of PPN neurons, only those neurons located within the cholinergic borders of the PPN (either in the same section or adjacent sections) were included in this study.

Neurons from the *in vitro* experiments were filled with biocytin during the electrophysiological recordings. The slices containing the filled neurons were fixed overnight (4% paraformaldehyde in 0.1M phosphate buffer; pH 7.4; 4°C). Permeabilization was achieved by incubation in Tris buffered saline (in mM, Tris base, 8; Trisma HCl, 42; NaCl, 150; pH 7.4) supplemented with 0.1% Triton X-100 and 10% bovine serum (60 min). The slices were incubated in phosphate buffer containing streptavidin-conjugated Alexa488 (1:300; Molecular Probes Inc., Eugene, OR, USA) for 90 min. The cells were visualized using a Zeiss LSM 510 confocal microscope (Carl Zeiss AG).

### Electrophysiological Data Analysis

Probe recordings of 300 s duration were manually selected to contain sustained SWA (15 epochs, length: 122.576 ± 62.225 s), sustained AS (10 epochs, length: 87.665 ± 35.515 s), and transitions between SWA and AS (9 epochs, length: 92.868 ± 15.568 s) based on the frequency and relative amplitude of the ECoG. Spike sorting of probe recordings was performed manually using Spike2 (CED, Spike2- 7.1). First, probe recordings were band pass filtered between 500 and 5000 Hz and channels with candidate units were selected. Only units with a signal-to-noise ratio above 2 and a consistent waveform across consecutive recordings were considered for further analysis. Then, spike trains of single units were detected based on the captured waveform, clustered according to features selected via Principal Component Analysis using in-built clustering algorithms in Spike2, and stored in spike train channels. Single units with inter-spike intervals below 2 ms were discarded.

In order to evaluate responses of PPN neurons to brain state transitions, spike trains were analyzed for changes in firing rate before, during and after sensory stimulation (hind paw pinch). Thus, the baseline of spontaneous unit activity, in terms of the mean firing rate prior to the onset of the stimulation, was compared to the activity both during, and immediately after, the stimulation. Similarly, changes in the regularity of firing in terms of coefficient of variation of firing were evaluated and compared before, during, and after sensory activation.

Coupling of single units to global (i.e., ECoG) and local (i.e., local field potentials, LFP) oscillations was evaluated using custom routines run in Matlab. Analysis of coupling to global activity was based on the ECoG channel or, for local coupling, on a fixed succession of five probe channels across the dorsoventral axis of the PPN. Wide band oscillations were filtered into the delta (0.3–3 Hz), theta (3–8 Hz), alpha (8–12 Hz), beta (12–30) and gamma (30–90 Hz) ranges (band-pass, finite impulse response with zero-phase delay with *Q* = 10, 10, 8, 8 and 8, respectively). Then, the instantaneous phase was computed by using the Hilbert transform. Phase histograms for each frequency were extracted from the instantaneous phases at the spiking times of PPN neurons. We estimated the circular mean of the phase distributions and assessed their significance according to the Rayleigh test for non-uniformity of circular data (Fisher, 1993). A false discovery rate threshold *p*_FDR_ < 0.05 was used in order to correct for multiple comparisons along the whole set of neurons, frequencies and states (Benjamini and Hocherg, 1995). Preferred phase of coupling were obtained by fitting the phase distribution to a von Mises distribution. Correlation between the preferred phase and strength of coupling was assessed by means of the correlation coefficient between one circular and one linear random variable.

Network activity was evaluated by the connection probability, defined as the number of significant spike-spike interactions relative to the number of all possible pairs of active PPN neurons. Two neurons were considered to interact if they showed a significant level of coherence (weighted periodogram estimate of coherence for two point processes; Halliday et al., 1995; Nielsen et al., 2005) within any of the frequency ranges of interest (*p* < 0.05).

Electrophysiological properties of PPN neurons were further characterized by analyzing their responsiveness to the sensory stimulation. Neurons were classified as phasically-excited, tonically-excited, inhibited or non-responding, depending on the changes detected in their instantaneous firing rate as a result of the stimulation. For this purpose, each spike train *k* was considered as a sequence of discrete events occurring at time points {*t_i_*}, *i* = 1, 2, … , *n_k_*. Adding up the number of events prior to and up to *t_i_*, defines a cumulative distribution function (CDF) at each time *t_i_* that is a strictly increasing step function changing at *t_i_* in unit steps with slope equal to the density of events per unit time. Computing a local linear regression of this CDF function based on six neighboring events gives the regression slope for each spike time and thus allows an estimation of the instantaneous firing rate, *FR_k_* (*t_i_*) for each train *k* at each reference time *t_i_* (Blejec, 2005).

Significant differences in the firing rate around the stimulation were evaluated by comparing the distributions of the instantaneous firing rates within the following periods: −15–0 s (baseline), 0–15 s (stimulation) and 15–30 s (post-stimulation; stimulation onset at 0). Instantaneous firing rates within the three periods were compared in pairs by computing the approximate bootstrap distribution of the difference of means (500 repetitions, *CI* = 95%; Efron, 1979). Neurons with no differences between the stimulation and the baseline/post-stimulation periods were classified as non-responding. Neurons with significantly lower instantaneous firing rates during the stimulation and post-stimulation periods compared to the baseline were assigned to the inhibited category. Neurons with higher instantaneous firing rate during the stimulation that did not differ between the baseline and post-stimulation periods were assigned to the phasic excitation category. Neurons showing significantly higher firing rates during the stimulation and post-stimulation periods compared to the baseline were assigned to the tonic excitation category.

Latency of the neuronal responses to the stimulation were investigated on the basis of the distribution of the instantaneous firing rate during the baseline. Percentiles 5 (*C_5_*) and 95 (*C*_95_) of the *FR(t)* during the basal period were selected as threshold parameters to assess the presence of inhibition or excitation responses within the stimulation or post-stimulation periods. Significance of response periods was assessed by means of a cluster-based permutation test (*n* = 200 permutations, *p* < 0.05) on the duration of the periods. If *FR(t)* during stimulation or post-stimulation was above percentile 95 or below percentile 5 for a longer period than any other within the baseline (and not less than 1 s), then the neuron was considered to respond. Finally, the first point of the first response period relative to stimulation onset was taken as the latency of the neuron response.

Together with changes in the firing pattern of PPN neurons, the stimulation elicited a drastic effect on cortical activity which defined the transition from SWA to AS. In order to detect the latency of such transitions, we first quantified the changes in the dynamics of the ECoG by estimating the approximate entropy (ApEn), a statistical measure that quantifies the unpredictability of fluctuations in a time series (Pincus, 1991). We computed the ApEn of the ECoG signal in overlapping (95%) windows of 2 s length around the stimulation (−30–45 s). Different values for the embedded dimension *m* (*m* = 2, 3 and 4) and tolerance parameter *r* (*r* = 0.1, 0.2 and 0.3 times the standard deviation of the ECoG signal) were tested. Best results were obtained for values are *m* = 4 and *r* = 0.2 times the standard deviation of the ECoG signal. The distribution of the ApEn values during the baseline was then used to detect activity changes as a consequence of the stimulation. Percentiles 5 (C_5_) and 95 (C_95_) of the ApEn estimate during the baseline were selected as thresholds to assess differences during the stimulation and post- stimulation periods. Significance of these periods was assessed by means of a cluster-based permutation test (*n* = 200 permutations, *p* < 0.05) on the duration of the periods. The latency of the cortical transition was set to the first time point corresponding to the first cluster with a significant change.

For the neurons recorded *in vitro*, the adaptation index (AI) was calculated from the 10 s long traces using the following formula: *AI* = 1−(F_last_/F_init_), where the F_last_ is the frequency of the last two action potentials of the trace, and F_init_ is the frequency of the first three action potentials (Nigro et al., 2014). To further investigate the frequency adaptation of PPN neurons *in vitro* and correlate their dynamics with the spike trains observed *in vivo*, we modeled the instantaneous firing pattern during sensory stimulation (*in vivo*) or current injection (*in vitro*) by a single decaying exponential function. To do this, the instantaneous firing rate of each neuron was fitted to a function *FR*(*t*) = *ae*^−τ*t*^ where *a* represents the firing rate at the beginning of the stimulation and *τ*, the adaptation exponent, models the decay of the firing frequency and thus models the degree of frequency adaptation of the neurons.

### Statistical Data Analysis

Differences in firing rates and regularity of single units across different states were tested with paired-sample Wilcoxon signed rank test. Differences between expected and observed frequencies in the number of neurons coupled to the global and/or local oscillations were assessed by using a Chi-square test with Yates’ continuity correction. Differences in the frequency adaptation were investigated by means of a two-way ANOVA test with factors: recording preparation (*in vivo*/*in vitro*) and neurochemical type (cholinergic/non-cholinergic). Multiple comparison test were applied when needed. All data are represented as mean ± CI, unless otherwise stated. The level of significance was set to *p* < 0.05.

## Results

### PPN Neurons Display Brain State-Dependent Discharge Properties

**Figure 1:**
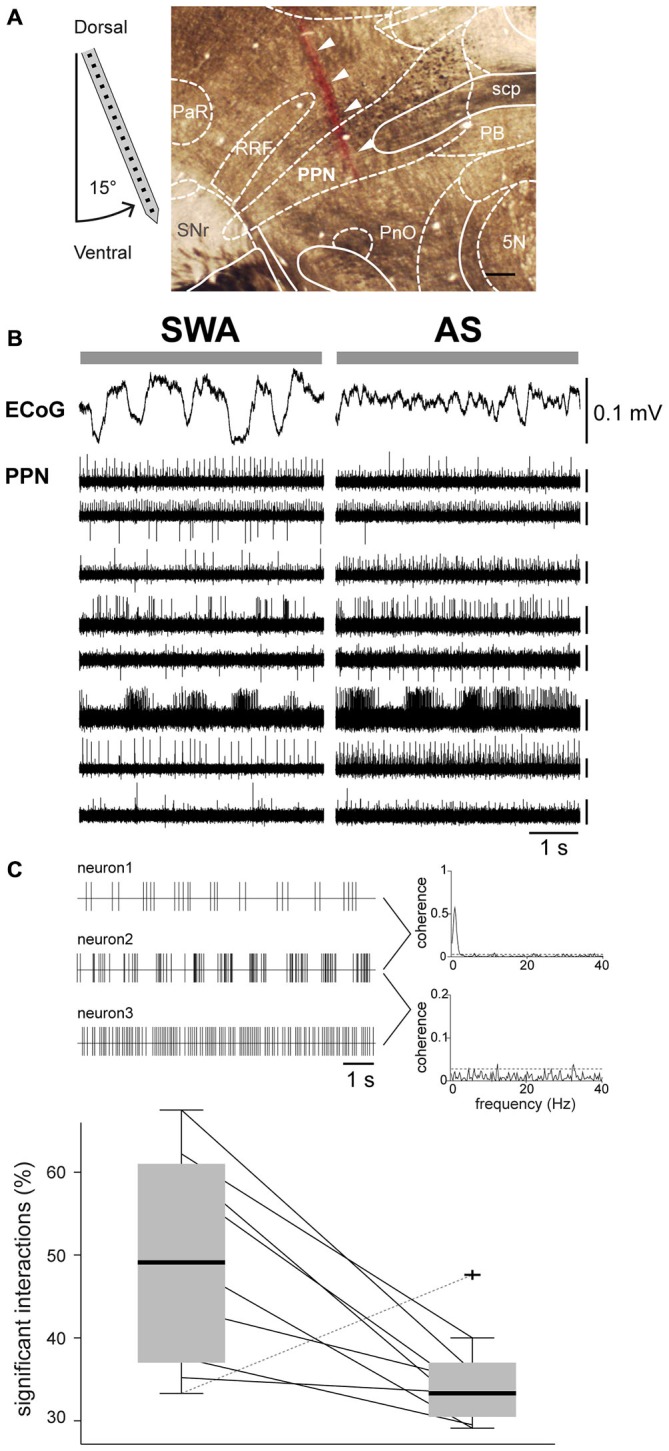
**Activity of PPN neurons is state dependent. (A)** Neurons were recorded in the dorsoventral axis with a 16- or 32-channels probe in the PPN (arrows indicate the probe track labeled with DiI). 5 N, motor trigeminal nucleus; PaR, pararubral nucleus; PB, parabrachial nucleus; PnO, pontine reticular nucleus, oral part; RRF, retrorubral field; SNr, substantia nigra pars reticulata. Scale bar: 300 μm. **(B)** High-amplitude and low-frequency activity was recorded in the ECoG during SWA, which was replaced by faster frequency activity and an “activated state” (AS). The signal across the PPN was filtered to extract multiunit activity, which was followed during both brain states. We detected highly heterogeneous activity across the whole extent of the nucleus. Scale bars apply to all traces. **(C)** During SWA, PPN neurons have a large proportion of significant interactions, indicative of high levels of network synchrony (illustrated in the representative traces as examples of coherent [top] and non-coherent [bottom] neuronal activity; dotted lines denotes significance levels), that decrease drastically during the AS in all cases but one.

The *in vivo* firing properties of PPN neurons were analyzed during two distinct brain states detected in the ECoG: SWA, consisting of predominantly delta activity and alternating UP- and DOWN-states (~1 Hz), and the AS, consisting of low-amplitude/fast-frequency activity and the obliteration of UP- and DOWN-states. Stable AS recordings (>40 s) were selected from either spontaneous occurrences or at least 2 min following sensory stimulation (hind paw pinch). Spike-sorted trains of neuronal activity were recorded from the PPN across the dorsoventral axis using high-density silicon probes (Figure 1A). We observed a wide range of firing rates among PPN neurons during SWA, ranging from 0.0139–43.2 Hz (*n* = 199), that were independent of their location within the PPN (Figure 1B, left). During the AS, the firing of PPN neurons ranged from 0.028–48.16 Hz (*n* = 116). A large proportion of these neurons were recorded in both states (*n* = 110) and showed a significant increase in the firing rate from SWA to AS (Wilcoxon signed rank test, *p* = 0.00003; median_SWA_ [Q_0.05_, Q_0.95_] = 1.3872 [0.0829, 21.6152], median_AS_ [Q_0.05_, Q_0.95_] = 2.3173 [0.1740 21.2992] Hz) and a significant decrease in their coefficient of variation (Wilcoxon signed rank test, *p* = 0.0018; median_SWA_ [Q_0.05_, Q_0.95_] = 0.9061 [0.3342, 2.0876], median_AS_ [Q_0.05_, Q_0.95_] = 0.8668 [0.2837, 1.5514]; Figure 1B, right). From these neurons, 69% increased and 31% decreased their firing rate during the AS. Thus, PPN neurons discharge differently across brain states with a large variability in the firing rates and their changes across states.

Next, we analyzed the network activity in the PPN by looking at multiple neuronal interactions in both brain states, SWA and AS. We obtained the connection probability by calculating the number of significant spike-spike interactions between each recorded PPN neuron and its neighbors across different temporal scales. During SWA we detected a significantly higher proportion of interactions compared to the AS (Wilcoxon signed rank test, *p* = 0.0273, mean SWA: 0.5 ± 0.13%; mean AS: 0.35 ± 0.06%). In all but one animal, PPN neurons that were firing simultaneously during SWA decreased the probability of interactions during the AS (Figure 1C). Thus, PPN neurons overall fire faster during the AS but their firing is not organized, as evident from their low level of interaction with neighboring neurons.

### Highly-Structured Temporal Organization of PPN Activity During SWA is Fragmented During Cortical Activation

**Figure 2:**
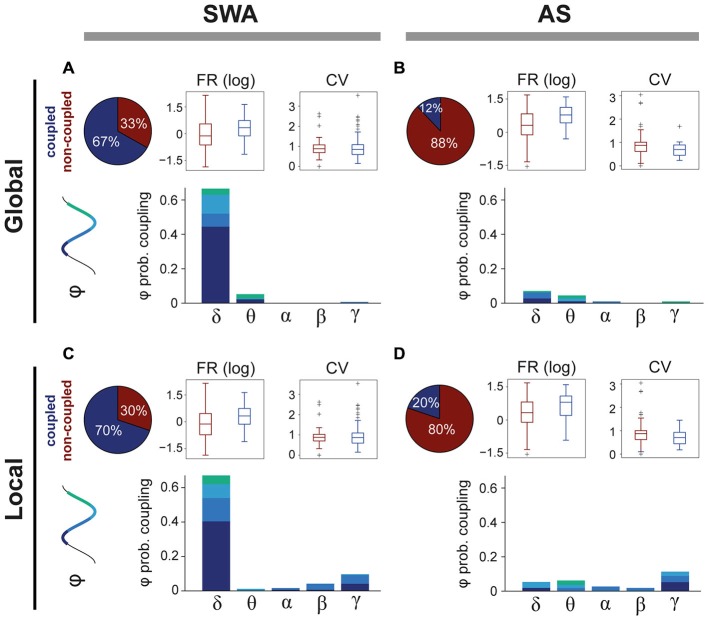
**Differences in coupling to oscillatory activity across brain states. (A)** The majority of PPN neurons couple to global oscillatory activity during SWA, predominantly during the peak of the slow oscillation (delta). The firing rate (FR) of coupled neurons is significantly higher (see text for details) than non-coupled neurons, but no differences in the coefficient of variance (CV) were detected. **(B)** In contrast, during the AS, only a small proportion of neurons remain coupled to global oscillations and their FR is significantly higher than non-coupled neurons (see text for details). **(C)** Similar results were observed for the coupling with local oscillations, when even a larger proportion of neurons are coupled to LFPs. **(D)** During the AS, PPN neurons that were coupled during the SWA become uncoupled. Some neurons couple to local fast-frequency oscillations during both SWA and AS, but they represent a small proportion.

SWA has a strong influence over the activity of some PPN neurons, but the extent of this modulation across brain states is not known. We analyzed the spike timing of PPN neurons (*n* = 205) in relation to global (from the frontal cortex) and local (from the local field potentials) oscillations (0.1–80 Hz) during SWA and the AS. The power spectra of both global and local oscillations was then decomposed to identify the predominant frequencies that modulate the firing of PPN neurons. We detected that during SWA the majority of PPN neurons couple to global (Figure 2A, upper left panel) and/or local (Figure 2C, upper left panel) frequency oscillations. As expected, during SWA most coupled PPN neurons were associated with oscillations in the largely predominant delta frequency range (66.3%), and particularly aligned around the slow oscillation (0.8–1.2 Hz; Figure 2A, lower panel). In agreement with our previously published data (Mena-Segovia et al., 2008), neurons coupled to the global slow oscillation are primarily modulated by the peak of the oscillation (UP state, 44.2%), while smaller proportions of neurons are modulated by the trough (DOWN state, 11.1%) or the transitions from peak to trough (7.5%) or trough to peak (3.5%); in addition, a fraction of neurons showed coupling with theta (5%) and gamma (0.5%). Similar to the global modulation, PPN neurons are predominantly associated with slow oscillations recorded locally (Figure 2C). In fact, most of the neurons coupled with the global slow oscillation are also modulated by the local slow oscillation (90.15%). However, in contrast to the global modulation, a fraction of PPN neurons was also coupled locally to faster frequency oscillations (16%), in particular to gamma oscillations (9.5%; Figure 2C, lower panel). Thus, during SWA, PPN neurons couple to both local and global oscillations mainly in the slow frequencies and to similar phases of the oscillation. The correlation coefficient between the preferred phase of coupling and the strength of interaction revealed that, during SWA, the strength of coupling results larger at the peaks and troughs of the local/global delta oscillations than at the other phases of the oscillation (*r* = 0.306, *p* = 0.002 and *r* = 0.360, *p* = 0.0002, respectively). No significant correlations were observed for the other frequencies.

During the AS, we detected a significant reduction in the number of PPN neurons coupled to global (Figure 2B; upper left panel) and/or local (Figure 2D, upper left panel) oscillations, compared to SWA (Chi-square test with Yates’ continuity correction, ${\text{χ}}_{1,315}^{2}$ = 69.83, *p* < 0.0001). The neurons that fired in phase with global oscillations during the AS were coupled to the same residual frequencies that they coupled to during SWA (delta 6.9% and theta 4.3%; Figure 2B, lower panel) but in significantly lower proportions, suggesting that the obliteration of the delta frequency *releases* the global coupling of most neurons and it is not replaced by a coupling with the frequencies associated to the AS (>4 Hz). A similar change occurs in relation to the local oscillations during the AS, although a slightly larger proportion of neurons remain coupled to the oscillations, particularly in theta (6%) and gamma (11%), although these represent a small minority (Figure 2D, lower panel). Thus, and although a significant degree of correlation between the preferred phase and strength of coupling was detected for the global delta (*r* = 0.972, *p* = 0.023) and local theta oscillations (*r* = 0.937, *p* = 0.046), the most evident change between brain states is a *decoupling* from both global and local oscillations, indicating that the coupling of PPN neurons to oscillations is state-dependent and that during the AS there is no structure in the oscillatory firing of PPN neurons.

In order to resolve whether the coupling to the oscillatory activities determine the firing rate or coefficient of variance of PPN neurons, we compared these values between coupled and non-coupled neurons in relation to the brain state (i.e., SWA vs. AS; Figures 2A–D). We found that neurons coupled to the local and global oscillations have significantly higher firing rates (Wilcoxon rank sum test for equal medians, *p*_(SWA, global)_ = 0.0004, *n*_coupled_ = 133, *n*_uncoupled_ = 66; *p*_(SWA, local)_ = 0.0004, *n*_coupled_ = 139, *n*_uncoupled_ = 60; *p*_(AS, global)_ = 0.0138, *n*_coupled_ = 14, *n*_uncoupled_ = 102; *p*_(AS, local)_ = 0.0391, *n*_coupled_ = 23, *n*_uncoupled_ = 93). On the other hand, no significant effects of the coupling are found for the CV, independently of the reference origin of the oscillations or state. Thus, coupling to global or local oscillations is associated with systematic differences in the discharge rate of PPN neurons.

### Neuronal Dynamics in the PPN During Brain State Transitions

**Figure 3:**
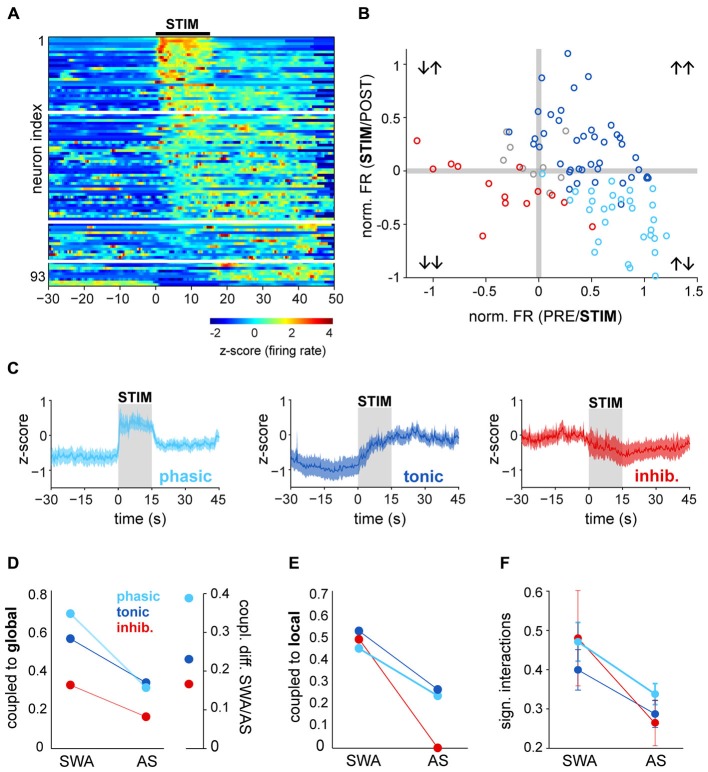
**Brain state transitions are associated with different responses in PPN neurons. (A)** Normalized firing rate (*z*-score along the whole recording) for each PPN neuron around the time of sensory stimulation (hind paw pinch; black bar, “STIM”) and sorted according to the latency and magnitude of their response to the stimulus. White lines represent the categories described in **(B,C)**. **(B)** Ratio of the changes to the stimulation comparing the firing rates during the baseline (“PRE”) vs. during the stimulation (*X* axis) and during vs. after (“POST”) the stimulation (*Y* axis). For each neuron, differences were first assessed by bootstrapping the instantaneous firing rate within each period and evaluating the bootstrap distribution for the mean difference. Neurons were categorized, color-coded and plotted depending on the significance of the PRE/STIM and STIM/POST changes in the firing rate. For presentation purposes, the mean differences were normalized by the mean firing rate of each neuron. **(C)** Normalized firing rate of the neurons that were grouped according to the categories detected in **(B)** showing three main dynamics of response to the stimulation: phasic activation, tonic activation or inhibition. **(D)** Proportion of neurons from each category (phasic, tonic, inhibited) that were coupled to the global oscillations during SWA and AS (left), and proportion change between brain states, revealing the largest variation for phasic neurons (right). **(E)** Proportion of neurons coupled to the local oscillations. **(F)** Proportion of significant interactions. Color codes in **(E,F)** as in **(D)**; data expressed as decimals.

PPN neurons become activated during transitions from slow-wave sleep to wakefulness (Steriade et al., 1990); as a result, they have been proposed to contribute to the maintenance of the waking state. Our results above, however, show that during a stable AS, PPN neuronal activity has a low level of synchronization and is decorrelated from both local network activity and global oscillations. In order to investigate further on the contributions of PPN neurons to the AS, we then analyzed PPN network dynamics at the time when the transition from SWA to AS occurs. To induce transition to the AS, sensory stimulation was delivered to the rat hind paw during stable SWA and the responses in the spiking activity of PPN neurons were analyzed. The instantaneous firing rate of individual spike trains was calculated around the time of stimulation (from −30 to 45 s, stimulation duration: 15 s) and the neuronal responses were categorized as excited (above the 95th percentile of firing rate before stimulation for a significant period of time) or inhibited (below 5th percentile). The majority of PPN neurons responded to the stimulation (excited: 74%; inhibited: 15%; no change: 11%; *n* = 93), although with a large variability in their latencies and the magnitude of the response (Figure 3A). Furthermore, such variability was more evident in excited neurons: while some neurons responded robustly but transiently to the stimulation (31%), a larger proportion was also excited and remained activated for a longer time (43%; Figure 3B). Based on their firing rate during the post-stimulation period, we categorized them as “phasic” when neurons returned to baseline values after the stimulation ceased, and “tonic” when neurons remained at significantly higher frequencies in the post-stimulation period compared to the baseline (Figure 3C). Inhibited neurons formed a single category. Thus, PPN neurons could be classified into three distinct categories based on their response properties: they were either excited or inhibited during brain state transitions, and their excitatory responses showed phasic and tonic dynamics.

Once we identified the dynamic attributes of PPN neurons during brain state transitions, we sought to define their level of integration into the local network and their modulation by global activity. Phasic neurons were more likely to be coupled to global SWA than tonic neurons and most notably, than inhibited neurons. They also represented the largest number of neurons that lost their coupling during the AS; i.e., they exhibited the most marked change in coupling between states (Figure 3D). Similar reductions in the local coupling (Figure 3E) and significant interactions (Figure 3F) were observed for all three types of neurons during the AS. These results show that phasic neurons are more strongly coupled to the global SWA than tonic or inhibited neurons, but during the AS none of them are able to preserve their structured activity. On the other hand, brain state transitions pull together their discharge responses into three distinct functional subclasses and produce a transiently coordinated response that is more likely to exert an impact on their targets.

### Differential Involvement of PPN Neurons in Brain State Transitions are Correlated to their Neurochemical Phenotype

**Figure 4:**
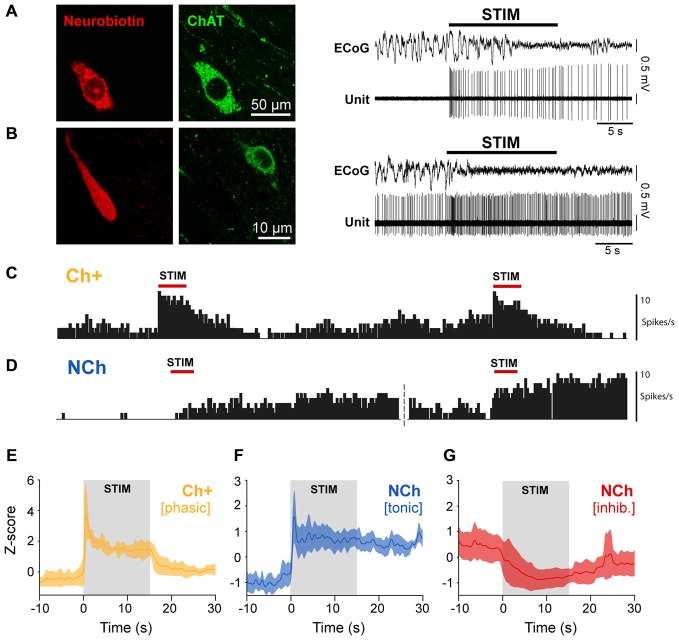
**The neurochemical properties of PPN neurons define their dynamics of activation. (A)** Neurons that were recorded and labeled *in vivo* (neurobiotin), and subsequently identified as immunopositive for choline acetyltransferase (ChAT), responded transiently to the sensory stimulation (representative trace). **(B)** In contrast, neurons that were immunonegative for ChAT showed prolonged responses to the same stimulation (representative trace). **(C)** Repeated stimulation trials show that cholinergic neurons rapidly return to a lower firing rate and respond in similar magnitude to successive stimulations. **(D)** Non-cholinergic neurons are able to maintain a steady and elevated firing rate after one trial, and further increasing their firing rate with successive stimulations. **(E)** Normalized firing rate of cholinergic neurons show a phasic dynamic of activation (*n* = 4). **(F)** Normalized firing rate of non-cholinergic neurons that were excited by the stimulation show a tonic dynamic of activation (*n* = 11). **(G)** Normalized firing rate of non-cholinergic neurons that were inhibited by the stimulation (*n* = 10).

In order to test whether such differences in the neuronal dynamics during global activation are correlated with the different cell types in the PPN, we analyzed the firing properties of PPN neurons that were recorded individually and labeled by the juxtacellular method during brain state transitions. Only neurons that responded to the stimulation were considered for this analysis. We found that all phasic neurons were positive for ChAT, and thus classified as cholinergic (Figure 4A), whereas all tonically excited and inhibited neurons were negative for ChAT (Figure 4B), and thus classified as non-cholinergic. The activation of phasic cholinergic neurons showed a robust immediate response that was restricted to the stimulation period and rapidly returned to baseline levels (Figure 4C), thus showing very similar dynamics (Figure 4E) to phasic neurons from the spike-sorted probe recordings. In contrast, the firing of tonic non-cholinergic neurons remained elevated after the stimulation and was capable of increasing to even greater values following subsequent stimulations (Figure 4D), distinctively showing higher firing rates than the baseline during both stimulation and post-stimulation periods (Figure 4F). Inhibited non-cholinergic neurons showed lower firing rates to the basal period during both stimulation and post-stimulation periods (Figure 4G). These results demonstrate that the phasic activation of cholinergic neurons is only sustained during sensory stimulation and cortical state transitions, but quickly return to their basal level of activity. In contrast, excited non-cholinergic neurons remain tonically activated beyond the stimulation, in line with the notion of a cell group in the PPN associated with maintaining a tonic level of excitation. Since some of these non-cholinergic neurons (*n* = 3) were used in previous studies and were shown to make asymmetric (excitatory) synaptic contacts in their target regions (Mena-Segovia et al., 2008; Ros et al., 2010), it is likely that these neurons are glutamatergic.

### Cooperative Mechanisms Between PPN Populations

**Figure 5:**
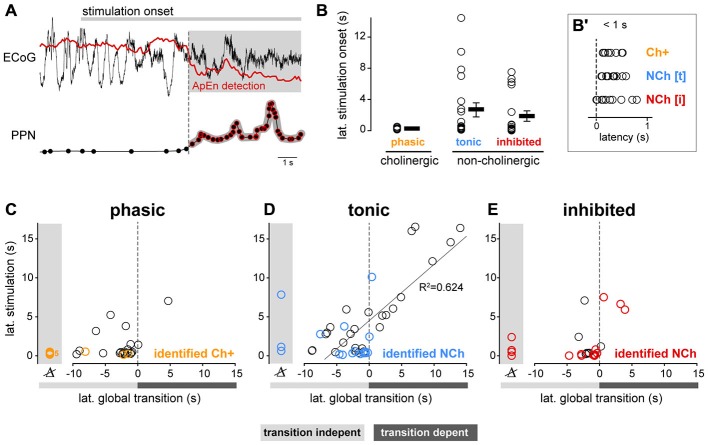
**Functional properties of PPN neurons correlate with latency during global brain transitions. (A)** The timing of the change in the spike trains of PPN neurons in response to the stimulation was extracted and correlated with the transition time using approximate entropy detection (ApEn). **(B)** Latency of the change in neuronal firing following sensory stimulation in neurochemically-identified neurons during different trials. While both cholinergic (*n* = 4) and non-cholinergic neurons (excited, *n* = 11; inhibited, *n* = 10) show short latency responses to the stimulation (inset, **B^′^**), only non-cholinergic neurons show longer latencies. **(C)** Phasic neurons show short-latency responses that precede the brain state transition, suggesting that their change in firing rate is independent of the global change in state; no significant correlation between their latencies was observed (*R*^2^ = 0.155, *p* = 0.063). **(D)** Tonic neurons showed early and late responses that were linearly correlated with the timing of the brain state transition (*R*^2^ = 0.735, *p* < 0.001). **(E)** Inhibited neurons responded predominantly before the transition; no correlation between their latencies was observed (*R*^2^ = 0.0932, *p* = 0.42). Linear regressions in **(C–E)** were calculated with data from silicon probes only (data points shown in black), whereas for neurochemically-identified neurons more than one stimulation trial is shown to illustrate the variability in their responses.

Both phasic cholinergic and tonic, putative glutamatergic neurons, seem to be coordinated during brain state transitions by producing excitatory responses but with different dynamics. To further analyze their response dynamics, we next evaluated the latencies of the changes in their spike trains following state transitions using both datasets. In order to do this, we identified the points in time when the cortical activity shifts from SWA to AS, thus revealing a change in the global brain state, and correlated them with the time points when the spike trains of PPN neurons (both neurochemically identified and from spike-sorted probe recordings) produce significant excitatory or inhibitory responses (Figure 5A). Following the stimulation onset, phasic cholinergic and subsets of both tonic and inhibited non-cholinergic neurons show significant changes in their firing rate with a short latency (<1 s; Figure 5B), suggesting a collective early role in brain state transitions. We observed that virtually all phasic PPN neurons (from probe recordings) and neurochemically-identified phasic cholinergic neurons, changed their firing rate *before* the change in the global brain state (i.e., transition-independent activation; Figure 5C), even in cases where the stimulation was not able to evoke a global transition. In contrast, tonic PPN neurons (from probe recordings) and neurochemically-identified tonic non-cholinergic neurons, showed both early and late responses to global brain state transitions, suggesting a dependency of their activation on the change in the global state in a proportion of them (Figure 5D). Finally, and similar to phasic neurons, inhibited neurons predominantly showed a transition-independent response, suggesting an early involvement in brain state transitions (Figure 5E). Thus, phasic cholinergic neurons respond with a short latency to sensory stimulation, preceding the cortical transition from SWA to AS, whereas non-cholinergic neurons display both short and long latency responses. Furthermore, the long latency responses in subsets of non-cholinergic neurons are associated with the cortical transition from SWA to AS, suggesting that the response of these neurons is dependent on the global environment. While the overall differences between PPN subtypes are likely to be the result of a balance between their distinct intrinsic properties and their synaptic inputs, the dynamic attributes during brain state transitions (i.e., phasic vs. tonic) may at least be partially explained by the former.

### Discharge of Cholinergic Neurons Show Rapid Adaptation

**Figure 6:**
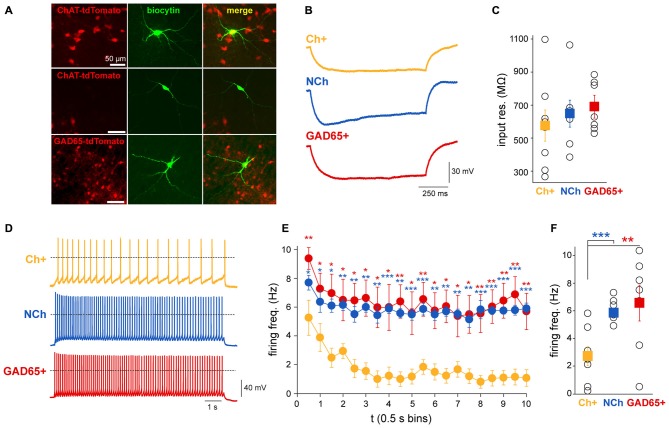
**Cholinergic neurons display a more pronounced firing frequency adaptation than GABAergic or other non-cholinergic neurons. (A)** Neurons that were recorded in the slice preparation in ChAT-tdTomato and GAD65-GFP mice were identified by fluorescence. **(B)** Representative voltage traces recorded with 1 s-long hyperpolarizing current injection (−30 pA) in a cholinergic, a GABAergic (GAD65+) and non-cholinergic neurons (NCh). **(C)** Input resistances of the recorded neurons. Although no significant difference was detected between the input resistances of the neuronal groups (see text for details), the average input resistance of cholinergic neurons is lower with a large variability. **(D)** Representative individual voltage traces from each subgroup. Dashed lines represent 0 mV. **(E)** Changes in the firing frequency during 10 s-long depolarizing current injections to −45 mV. Individual data points show the firing frequency in 0.5 s bins. Red asterisks indicate significant differences between data obtained from cholinergic and GABAergic neurons, while blue asterisks indicate significant differences between cholinergic and non-cholinergic neurons. **(F)** Differences of the overall firing frequency of a 10 s-trace. Squares represent the average ± SEM and hollow circles show the individual values (cholinergic *n* = 8, GABAergic *n* = 7 and non-cholinergic *n* = 8). *p < 0.005; **p < 0.01; ***p < 0.001.

In order to elucidate whether the different dynamics of PPN neurons observed during sensory stimulation and brain state transitions are related to their intrinsic physiological properties, the firing frequency adaptation of different neuronal populations was revealed in slice preparations. Whole-cell patch clamp recordings were performed on coronal midbrain slices from mice expressing tdTomato fluorescent protein associated to either the promoter of ChAT or glutamate decarboxylase 65 (GAD65) to identify either cholinergic or GABAergic neurons, respectively (Figure 6A). A sample of non-cholinergic (ChAT-) neurons were also obtained from the ChAT-tdTomato mice to cover the full spectrum of non-cholinergic neurons, which may include glutamatergic and GABAergic neurons. In order to determine their input resistance, a 1 s-long hyperpolarizing current injection of 30 pA amplitude was applied and potential changes were measured at the end of the current pulse (Figure 6B). Cholinergic neurons had an average input resistance of 578.5 ± 94.8 MΩ (range 267–1100 MΩ; *n* = 8), whereas GABAergic cells had an average input resistance of 693.4 ± 56.9 MΩ (range 530–887 MΩ; *n* = 7), and non-cholinergic neurons (ChAT-negative) displayed an average of 650.9 ± 81.6 MΩ (ranging from 387–1067 MΩ; *n* = 8; Figure 6C). No significant differences in input resistance were found between the neuronal groups (one-way ANOVA, *F*_2,19_ = 0.53, *p* = 0.5991). Next, 10 s-long depolarizing current injections were applied in 5 pA steps. These sweeps were compared when the neurons were depolarized to approximately −40 mV. The magnitude of the current injections necessary for this depolarization was between 20 and 75 pA. We found that the firing frequency in cholinergic neurons had significantly stronger adaptation than in non-cholinergic or GABAergic ones (Figure 6D), resulting in a significant difference in the adaptation index (*H* = 6.96, *p* = 0.031, ANOVA on ranks; cholinergic: 0.65 ± 0.12, non-cholinergic: 0.06 ± 0.09, GABAergic neurons: 0.18 ± 0.2; *post hoc* comparisons show significant differences between cholinergic and non-cholinergic neurons). At the beginning of the depolarizing current injection, cholinergic neurons fired with a frequency of 6.1 ± 0.9 Hz, whereas at the end of the current pulse this frequency dropped significantly to 1.7 ± 0.9 Hz (*p* = 0.002). In contrast, although non-cholinergic and GABAergic neurons also showed some adaptation, this was less pronounced compared to the cholinergic ones. The initial firing frequency of the GABAergic neurons was 9.4 ± 0.8 Hz which decreased to 5.7 ± 1.3 Hz (*p* = 0.01); and the firing frequency of non-cholinergic neurons at the beginning of the current injection was 7.7 ± 0.5 Hz and 5.9 ± 0.4 Hz at the end of the depolarizing pulse (*p* = 0.007; Figure 6E). Although this decrease in frequency was significant in for all subgroups, the magnitude of the decrease showed a significant difference in almost all time bins over the whole time of the stimulation. Furthermore, the firing frequency during the whole 10 s-long depolarizing impulse also differed significantly (one-way ANOVA, *F*_2,20_ = 5.09, *p* = 0.0177). Cholinergic neurons were different from GABAergic (*p* < 0.01) and from non-cholinergic neurons (*p* < 0.05), but there were no differences between GABAergic and non-cholinergic neurons (Tuckey *post hoc* comparisons). The overall firing frequency of the cholinergic neurons was 2.7 ± 0.7 Hz, for the GABAergic neurons was 6.6 ± 1.3 Hz, and 5.8 ± 0.3 Hz for the non-cholinergic neurons (Figure 6F). In two cholinergic and a single GABAergic neuron, transient activation was observed: these neurons only fired 2–4 action potentials at the beginning of the depolarizing current steps. These results show that cholinergic neurons rapidly adapt their spiking activity during continuous stimulation, whereas GABAergic and putative glutamatergic neurons remain firing at constant frequencies.

**Figure 7:**
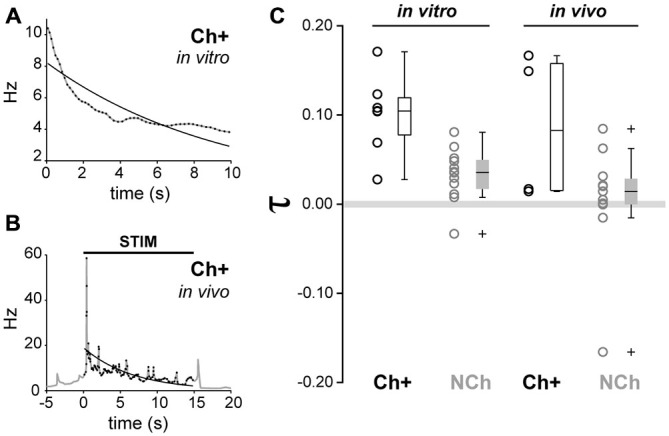
**Similar adaptation dynamics *in vivo* and *in vitro* in cholinergic neurons. (A)** Example of exponential fitting for a representative cholinergic neuron recorded *in vitro*. The decay of the instantaneous firing rate can be modeled by a single exponential function with adaptation exponent (*τ* = 0.1044). **(B)** Example of exponential fitting for an *in vivo* representative cholinergic neuron (*τ* = 0.1491). **(C)** Cholinergic neurons show a significantly higher degree of frequency adaptation than non-cholinergic neurons (circles represent individual data points, boxes represent median, quartiles 25/75 and percentiles 5/95). No significant effects due to the difference of preparation (*in vivo* vs. *in vitro*) nor interactions were detected.

Finally, in order to compare the firing rate adaptation observed from *in vitro* recordings with the firing dynamics observed *in vivo* following sensory stimulation, we calculated the adaptation exponent **τ**, which models the decay of the instantaneous firing rate across time, for all neurochemically-identified neurons that were recorded *in vivo* and *in vitro* (Figures 7A–C). From *in vivo* juxtacellular recordings, cholinergic neurons showed an adaptation exponent **τ** median [Q_0.05_, Q_0.95_] = 0.0828 [0.0142, 0.1665] Hz/s, whereas non-cholinergic neurons had an exponent closer to 0, thus denoting a firing pattern with a more regular rate (median [Q_0.05_, Q_0.95_] = 0.0143 [−0.1584, 0.0833] Hz/s). From the *in vitro* recordings, the exponent of cholinergic neurons was **τ** median [Q_0.05_, Q_0.95_] = 0.1044 [0.0276, 0.1709] Hz/s, whereas for non-cholinergic (both GAD+ and ChAT-) **τ** median [Q_0.05_, Q_0.95_] = 0.0357 [−0.0293, 0.0791] Hz/s (Figure 7C). A two-way ANOVA test (neurochemical type × modality of recording) detected significant differences only for the neurochemical factor (F_1,30_ = 13.33, *p* = 0.001). *Post hoc* multiple comparison tests revealed that cholinergic neurons exhibited stronger frequency adaptation than non-cholinergic neurons (*p* < 0.05). No significant effect of the recording paradigm (*in vivo* vs. *in vitro*, *F*_1,30_ = 2.6, *p* = 0.0637) nor of the interaction between factors was detected (*F*_1,30_ = 0, *p* = 0.949). Therefore, the evaluation of the exponent fitting the decay in the instantaneous firing rate reinforces the fact that cholinergic neurons show stronger adaptation than non-cholinergic neurons, in both *in vivo* and *in vitro* preparations. Thus, our *in vitro* characterization of adaption dynamics of PPN neurons are in line with a phasic role of cholinergic neurons in the PPN output during brain state transitions.

## Discussion

Our data describe the neuronal dynamics in the PPN during two distinct brain states, SWA and AS, and the transition between them. Using high-density recordings, we show that during SWA the activity of PPN neurons is highly structured, with the majority of them coupled to both global and local oscillations and acting in synchrony with neighboring neurons. In contrast, during the AS the activity of PPN neurons is not temporally organized: their discharge is unconstrained from network oscillations and dissociated from local neurons. Nevertheless, the majority of PPN neurons respond robustly to sensory stimuli that elicit transition between SWA and AS and display three main types of response: phasic excitation, tonic excitation or inhibition. Using juxtacellular recordings and subsequent labeling, we demonstrate that neurons that respond with phasic excitation are cholinergic neurons, whereas those neurons that maintain a tonic level of excitation that extends beyond the transition are non-cholinergic (putative glutamatergic). Furthermore, the *in vitro* intracellular recordings show that the spiking of cholinergic neurons adapts rapidly to a long current injection, in contrast to non-cholinergic neurons which maintain a constant firing with little adaptation. Our results demonstrate both complementary and divergent roles of PPN neurons during brain state transitions. Furthermore, our data challenge some of the long-held notions of the contribution of the PPN, and in particular of cholinergic neurons, to the modulation of the global AS.

### Limitations of Our Study

Our data is valuable to understand the functional interactions within neuronal circuits whose activity typically depends on the waking state. We interpret our data within the constraints of the use of anesthesia that was required to maintain a stable preparation for multisite/high-density recordings in different brain states and for juxtacellular recordings, and the possibility to correlate both datasets. We utilized the changes in the electrocorticographic activity to define global brain states during both induced and spontaneous activation. It has been shown by others and ourselves that both the structure and pattern (Clement et al., 2008) and the oscillatory components of SWA during urethane anesthesia (Valencia et al., 2014) closely resemble those of natural sleeping animals (Steriade, 2006). In the case of the AS, the firing rate of PPN neurons reported here are within the range of the rates reported in the awake rat during “quiet wakefulness” (Boucetta et al., 2014). Thus, the patterns and structure of brain activity during SWA and AS reported here closely resemble those of natural sleeping animals.

An additional caveat is the use of sensory stimulation to investigate brain state transitions. While we have previously shown that spontaneous transitions during urethane anesthesia are associated with increased firing in cholinergic neurons (Mena-Segovia et al., 2008), they tend to occur over several seconds, thus providing a poor temporal resolution to identify differences in the dynamics of subsets of PPN neurons. By using pinch stimulation, we were able to normalize the latencies across different animals and compare the data of a large pool of neurons. It is worth noting that while PPN neurons respond to different modalities of sensory stimulation (Pan and Hyland, 2005; Okada et al., 2009) that activate specific subsets of neurons, perhaps in a context-dependent manner (see below) and with different latencies, the signal generated by the pinch is likely overriding the functional connectivity with other systems in order to induce arousal. Indeed, pinch stimulation has been shown to induce acetylcholine release in the thalamus of urethane-anesthetized rats (Motelow et al., 2015), thus simulating the waking-related effects on thalamic cholinergic transmission (Paré et al., 1990).

### Contributions of the PPN to the Waking State and Arousal

Considered as a part of the reticular activating system (Moruzzi and Magoun, 1949), a subset of PPN neurons were reported to fire at a high frequency during wakefulness and to decrease their activity during sleep (El Mansari et al., 1989; Steriade et al., 1990; Sakai, 2012), contributing to the idea that the cholinergic brainstem is involved in the modulation and maintenance of wakefulness (for a review, see Saper et al., 2005). Here, we analyzed the temporal structure of the neuronal activity in the PPN during the AS. Surprisingly, we observed a lack of synchrony in the overall network dynamics in the PPN during stable AS, as indicated by the absence of neuronal coupling to oscillatory activity and a reduced level of local neuronal interactions. These results show that, during stable AS, PPN neurons fire in uncoordinated fashion, suggesting that their contribution to the AS is not oscillatory in nature and may not extend over the AS. Interestingly, lesions of the PPN in rats are not associated with major changes in the sleep-wake architecture nor with a decrease in wakefulness (Deurveilher and Hennevin, 2001; see review in Winn, 2006). The lesion studies also coincide with some of the features observed in progressive supranuclear palsy (PSP) patients, where degeneration of PPN cholinergic neurons play a major role in the disease (Mazère et al., 2012). While PSP patients recount impaired sleep efficiency, no changes in day sleepiness scores were observed (Gama et al., 2010), conflicting with the notion that cholinergic PPN neurons maintain the waking state. In summary, these findings raise questions as to the significance of the cholinergic PPN neurons in *maintaining* wakefulness.

Our results show that PPN neurons respond robustly to brain state transitions but have distinct dynamics. We identified two distinct types of excitatory responses associated with global activation: a phasic response, where the firing rate increases rapidly and robustly at the onset of the sensory stimulation and decreases progressively, returning to a lower rate immediately after the stimulus ceases, and a tonic response, which has a longer latency of activation but maintains a steady elevated discharge rate long after the stimulus is over. Our data from neurochemically-identified neurons show that those neurons giving rise to phasic responses are cholinergic neurons and those giving rise to tonic responses are non-cholinergic, possibly glutamatergic neurons. Thus the role of cholinergic neurons is transient and highly sensitive to the somatosensory context (in agreement with MacLaren et al., 2014), and may be modulated by other arousal-promoting systems (e.g., orexin; Ishibashi et al., 2015). In contrast, putative glutamatergic neurons may act in parallel with cholinergic neurons providing a stable activation beyond the brain state transition, suggesting that their pattern of activity may correspond to those originally believed to be cholinergic in experiments where the neurochemical phenotype could not be identified (El Mansari et al., 1989; Steriade et al., 1991; Datta and Siwek, 2002). Finally, inhibited non-cholinergic neurons (possibly GABAergic; see Boucetta et al., 2014) also respond with a very short latency to the stimulation, implying an early involvement in brain state transitions, possibly through the influence of local interactions. Thus, while our findings support the notion of cholinergic neurons as a part of a system that promotes arousal, they seem to have a less prominent role in sustaining a high cholinergic tone in their targets during wakefulness than previously believed (although see Williams et al., 1994) and most importantly, illustrate the transient dynamics of their activation.

### The Multifaceted Role of Cholinergic Neurons

Cholinergic neurons of the PPN and laterodorsal tegmental nucleus have been proposed to be involved in different functions, including attention, movement and reward. While it is widely assumed that cholinergic neurons are tonically active during wakefulness (Datta et al., 2001; Sakai, 2012; Urbano et al., 2014), the impact of cholinergic transmission on distinct functional systems is not compatible with a constant, spontaneously active structure. For example, optogenetic activation of cholinergic PPN axons evoke a phasic increase in the discharge of dopamine neurons (Dautan, unpublished observations); such an increase, in turn, is able to produce a temporally precise release of dopamine in their targets (Gonon and Sundstrom, 1996). Furthermore, PPN recordings in behaving rats and monkeys show a transient activation of PPN neurons in response to sensory events that evoke alertness or that elicit a behavioral response (Pan and Hyland, 2005; Okada et al., 2009; Norton et al., 2011; Thompson and Felsen, 2013; Hong and Hikosaka, 2014). While no neurochemical characterization was obtained to ascertain the cholinergic phenotype of the recorded neurons, all of the above studies show transient responses in PPN neurons that become activated in response to salient events and whose influence on their targets may shape the behavioral measures reported.

Here, we propose that the activation of cholinergic neurons is governed by salient events that elicit global activation and we demonstrate that the nature of this activation is transient. Such dynamics of cholinergic neurons are partially determined by their intrinsic properties which prevent them from firing tonically over extended periods, although the duration of the activation is likely to be contingent on distinct physiological contexts. Their high adaptation index would allow cholinergic neurons to be responsive to different modalities of salient events, thus supporting their role in attentional processes.

## Conclusion

PPN neurons show highly structured activity during SWA that recedes and is replaced by decorrelated activity during AS. The phasic and short-latency responses of cholinergic neurons to sensory stimulation suggest that they actively participate in the brainstem mechanisms that produce brain state transitions, while the slower but sustained responses of some non-cholinergic neurons suggest a role in the maintenance of the global AS. Our results show that a complex interplay between PPN neurons occurs during brain state transitions and propose to redefine the role of cholinergic neurons in the modulation of the waking state. The causality of the discharge of the different neuronal types for the modulation of the global brain state remains to be established.

## Author Contributions

JM-S conceived the project, designed and performed the *in vivo* experiments. BP performed the *in vitro* experiments. AP, MV, BP and JMS analyzed the data and wrote the article.

## Funding

This work was supported by the Medical Research Council UK (MC-UU-12020/1 to J. P. Bolam). BP was supported by a János Bolyai Research Scholarship of the Hungarian Academy of Sciences, a Szodoray Scholarship of the University of Debrecen and the National Brain Research Program (KTIA_13_NAP-A-I/10). MV was supported by a grant from de Departamento de Salud, Gobierno de Navarra (114/2014). Access to data will be available on request.

## Conflict of Interest Statement

The authors declare that the research was conducted in the absence of any commercial or financial relationships that could be construed as a potential conflict of interest.
